# Dewetting‐Assisted Interface Templating: Complex Emulsions to Multicavity Particles

**DOI:** 10.1002/advs.202203265

**Published:** 2022-08-12

**Authors:** Naresh Yandrapalli, Markus Antonietti

**Affiliations:** ^1^ Max Planck Institute of Colloids and Interfaces Department of Colloid Chemistry Am Mühlenberg 1 14476 Potsdam Germany

**Keywords:** complex emulsions, dewetting, microfluidics, multicavity particles, nanoindentation, polymersomes, proptose emulsions

## Abstract

Interfacial tension‐driven formation of intricate microparticle geometries from complex emulsions is presented in this work. Emulsion‐templating is a reliable platform for the generation of a diverse set of microparticles. Here, water‐in‐styrene‐in‐water complex emulsions undergo reproducible metamorphosis, i.e., from liquid state emulsions to solid structured microparticles are employed. In contrast to the traditional usage of glass‐based microfluidics, polydimethylsiloxane (PDMS) swelling behavior is employed to generate complex emulsions with multiple inner cores. In the presence of block copolymer surfactant, these emulsions undergo gravity‐driven dewetting of styrene, to transform into membranous structures with compartments. Further polymerization of styrene skeletal remains resulted in microparticles with interesting geometries and intact membranes. Mechanical and confocal microscopic studies prove the absence of polystyrene within these membranes. Using osmotic pressure, membrane rupture and release of encapsulated gold nanoparticles from such polymerized emulsions leading up to applications in cargo delivery and membrane transport are promoted. Even after membrane rupture, the structured microparticles have shown interesting light‐scattering behavior for applications in structural coloring and biosensing. Thereby, proving PDMS‐based swelling as a potential methodology for reproducible production of complex emulsions with a potential to be transformed into membranous emulsions or solid microparticles with intricate structures and multiple applications.

## Graphical abstract

A new methodology to produce materials of exceptional geometry is reported. Polydimethylsiloxane swelling and dewetting‐assisted interface templating is adopted as a strategy to generate skeletal frames from polymerizable complex emulsions produced using microfluidics. These new geometries from old‐school materials are envisioned to provide opportunities for the development of new structural and functional materials.

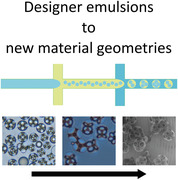

## 1 Introduction

Microfluidic‐assisted generation of emulsions in general and complex emulsions, in particular, gave birth to a new breed of structural and functional materials.^[^ ^1^ ^]^ Among many, core–shell structures are of great importance.^[^ ^2^, ^3^ ^]^ Not only can they be of exceptional material properties, the possibility to encapsulate materials for various applications is also exploited, extensively. We, along with other researchers, have used microfluidic double emulsion‐templating as a methodology to produce different modes of core–shell structures including soft lipid vesicles and hard polymeric microspheres.^[^ ^4^, ^5^, ^6^, ^7^ ^]^ However, structured microparticles or particles with cavities are different from traditional solid or hollow microspheres. Unlike the later, structured microparticles have unique geometries with associated applications.^[^ ^8^ ^]^ One prominent methodology is via the colloidal assembly with a series of steps involving assembly, templating, and sacrificial steps.^[^ ^9^, ^10^ ^]^ This multistep process is laborious whose reproducibility and homogeneity in producing multicavity microparticles are not streamlined. On the other hand, microfluidics represents reproducibility and precision control.

**Figure 1:**
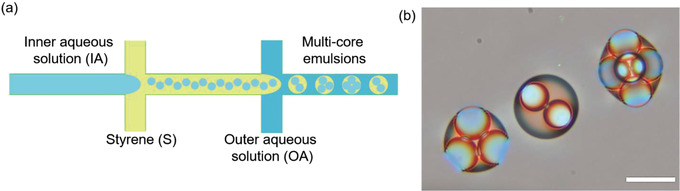
Generation of multicore emulsions. a) Schematic illustration of PDMS‐based microfluidic design used to produce multicore emulsions. b) True color images of multicore emulsions, left to right—3‐in‐1, 2‐in‐1, and 5‐in‐1 (scale bar: 50 µm).

In the present work, we attempted polydimethylsiloxane (PDMS)‐based microfluidics to achieve multicavity microparticles via dewetting and interface polymerizing of complex emulsions. A methodology reported earlier involves complex glass‐based microfluidic set‐ups and a lack of investigation into step‐wise transition and potential applications.^[^ ^11^, ^12^ ^]^ PDMS‐based microfluidics as well as glass microfluidics is widely used to produce double emulsions and associated microparticle derivatives.^[^ ^5^, ^6^, ^13^ ^]^ However, to produce multicavity microparticles, it is imperative to employ polymerizable multicore emulsions or double emulsions with multiple inner cores which are exclusively produced using glass‐based microfluidics, but not PDMS‐based.^[^ ^6^, ^11^, ^14^ ^]^ Polymerizable monomers such as styrene can alter PDMS microstructures via volumizing. A large set of solvents has been studied for their PDMS swelling properties but not for functional applications in droplet‐microfluidics and more specifically in complex emulsion generation.^[^ ^15^, ^16^, ^17^, ^18^ ^]^ In this work, we took advantage of PDMS swelling behavior to engineer multicore emulsions. A schematic of multicore emulsion production is presented in **Figure** **1**a along with color images of the emulsions produced in this work (Figure 1b).

Typical production of these emulsions involves the initial formation of water‐in‐oil droplets (W/O) at the 1st cross‐junction, followed by encasing more than one W/O droplet in aqueous media at the 2nd cross‐junction to yield water‐in‐oil‐in‐water (W/O/W) emulsions with varying inner aqueous cores. However, the usage of styrene presents a challenge in the form of PDMS swelling. The channel dimensions at the 1st cross‐junction should be large enough to not result in complete closure due to the swelling. Styrene used in the middle oil phase instantly constricts the 1st cross‐junction where W/O droplets are formed. This constriction leads to higher shearing of the water phase to produce smaller W/O droplets without needing to apply higher flow rates. Using this technique, we produced uniform size emulsions with multiple inner cores and further exploited the dynamic dewetting behavior of emulsions, in the presence of surfactant, to engineer new microparticle geometries.

Double emulsion‐templating of material synthesis is exclusively dependent on the interfacial forces and their stability, controlled by the type and the concentration of surfactant.^[^ ^6^, ^19^ ^]^ According to wetting theory, three interfacial tensions govern the equilibrium configuration of the emulsion, i.e., to transition from an excess oil phase containing emulsion to a minimal one—interfaced with surfactant membranes.^[^ ^19^ ^]^ The role of the density of the aqueous droplets (in the case of W/O/W emulsions) in assisting such a transformation is, in our opinion, hardly emphasized. While acorn‐like architectures are frequently observed during dewetting of 1‐in‐1 emulsions, such a transformation is not observed in the case of multicore emulsions. Instead, gravity‐assisted bilayer formation and dewetting associated with solvent evaporation resulted in a gradual transformation of multicore emulsions to protruded emulsion shapes. Further polymerization of the left‐over styrene within these protruded emulsion shapes resulted in compartmentalized microparticles with intact membranes. Interestingly, these microparticles are not only highly stable but also show robust mechanical properties deduced using atomic force microscopy (AFM)‐based nanoindentation. We have shown that the intact membranous windows along the microparticle frames can be ruptured to release cargo and yield final multicavity microparticles. These “frame architectures,” compared to simple droplets, have an increased surface area to volume ratio (SA:V) as the material is carved from the spherical emulsion. This methodology of producing multicavity microparticles is advantageous over other strategies, thanks to the precision control provided by microfluidics. Furthermore, the transition steps lead to different emulsion maturation stages, from a multicore emulsion to its protruded membranous emulsion to a polymerized alternative, finally into a structured solid multicavity microparticle. Even more, at any maturation stage, these microparticles have applications in selective permeability via membrane protein incorporation, cargo storage and release, and even biosensing and structural coloring possibilities. More interestingly, extrapolating the methodology by incorporating functional and polymerizable monomers will yield new materials with interesting properties and functionality.

## 2 Results

### 2.1 Dewetting‐Assisted Formation of Emulsion Templates

**Figure 2:**
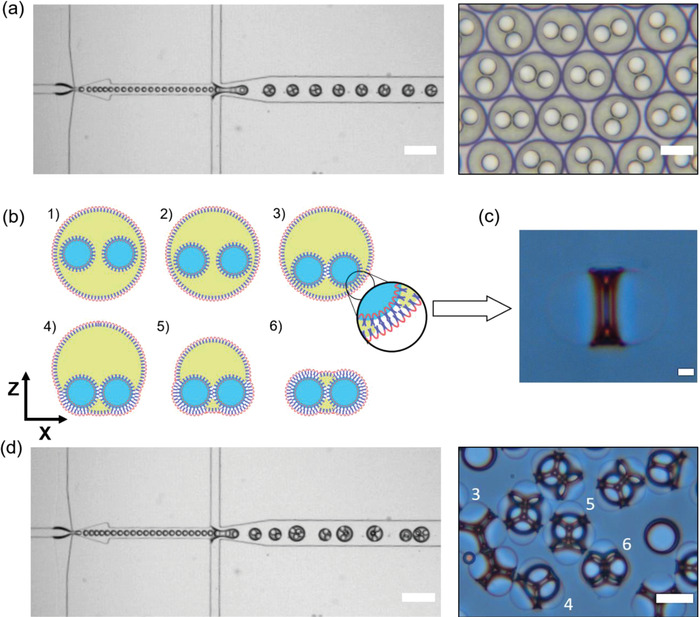
Multicore emulsions to Proptose emulsions. a) High‐speed camera visualization of 2‐in‐1 emulsion production (scale bar: 200 µm). Inset showing the microscopic images of the emulsions (>95% having the same number of inner cores) (scale bar: 50 µm). b) Schematic representation of gravity‐driven sedimentation of inner aqueous cores to assist in the formation of block copolymer membranes (1–3), followed by their gradual transformation (interfacial tension‐induced dewetting and solvent evaporation potentiates this transition) to proptose emulsions (3–6). c) Here shows a color image of the 2‐in‐1 proptose emulsion (scale bar: 10 µm). The gradual transition of multicore emulsions to proptose emulsions from left to right (scale bar: 50 µm). d) High‐speed camera visualization of multicore emulsions production with a varying number of inner cores. Inset showing the transitioned multicore emulsions into their proptose emulsions (scale bar: 50 µm).

PDMS‐based microfluidics was used to produce multicore emulsions with a varying number of inner aqueous cores. Deviating from the typical usage of glass‐based microfluidics to produce such complex emulsions, the swelling of PDMS in the presence of nonpolar solvents is exploited. It is well‐known that PDMS swells in contact with specific solvents.^[^ ^16^, ^17^ ^]^ While it is possible to produce small diameter emulsion droplets in microfluidic channels, it is not feasible (high‐pressure flow rates are required) when the oil phase (continuous phase) is less dense or low in viscosity compared to the water phase (disperse phase) as the shearing pressure required is high, thus high fluid flow rates. Pumping PDMS swelling solvents into the microfluidic chip with high pressures will not only block narrow channels, but we also observed peeling of PDMS from the glass surface. For this reason, we incorporated a PDMS design with wider channels, yet exploited the swelling properties of PDMS in the presence of styrene to yield microfluidic channels that can produce smaller W/O droplets, essential for multicore emulsion production. We have performed multiple PDMS swelling cycles (more than three) via sequential flooding of the microfluidic channels with either water or styrene. Snapshots of two swelling cycles performed within the same microfluidic chip can be seen in Figure S1a in the Supporting Information. The data from the measured dimensions are used to calculate the PDMS swelling degree (SD) value of 69 ± 13% for styrene, close to isopropanol.^[^ ^15^ ^]^ Overall, these results confirm reversible dimensional dilation of PDMS through styrene penetration and volume increase. The reversible nature of swelling can be attributed to pervaporation of styrene and lack of continuous supply when only water is being pushed through the microfluidic channels.^[^ ^16^, ^20^ ^]^ Further, time‐lapse snapshots in Figure S1b in the Supporting Information present a previously understood mechanism of droplet size reduction with lowering physical dimension (at constant fluid flow rates), in this case, due to PDMS swelling and constriction of the cross‐junction. Since the swelling of PDMS is finite (directly proportional to the degree of PDMS crosslinking), the swelling process reached its peak within 30 s with no further swelling as observed in a previous study that could potentially affect the polydispersity of the droplets and emulsions produced (Figure S1b, Supporting Information).^[^ ^15^ ^]^ Finally, this mechanism enables simple manipulation of fluid flow rates to generate and later encase multiple inner drops in the multicore emulsions. **Figure** **2**a represents such an effort to achieve multicore W/O/W emulsions with two inner aqueous cores encased in styrene. Careful calibration of fluid flow rates can not only generate a single emulsion type (2‐in‐1 in Figure 2a) but also simultaneous production of 2‐in‐1, 3‐in‐1, 4‐in‐1, and 5‐in‐1 multicore emulsion mixture (Figure 2d and Video S1, Supporting Information). A plot elucidating the relationship between fluid flow rates versus the number of inner cores encased in an emulsion is presented in Figure S1c in the Supporting Information. From the plot, it can be deduced that encasing inner cores to form multicore emulsions is inversely proportional to the flow rate of outer aqueous solution (*Q*_OA_) at a constant *Q*_IA_/*Q*_S_ ratio where *Q*_IA_ is the flow rate of inner aqueous solution and *Q*_S_ is the flow rate of styrene phase. Together with PDMS swelling data, the plot with hydrodynamic conditions to produce various types of complex emulsions presents a reliable PDMS‐based microfluidics methodology.

**Figure 3:**
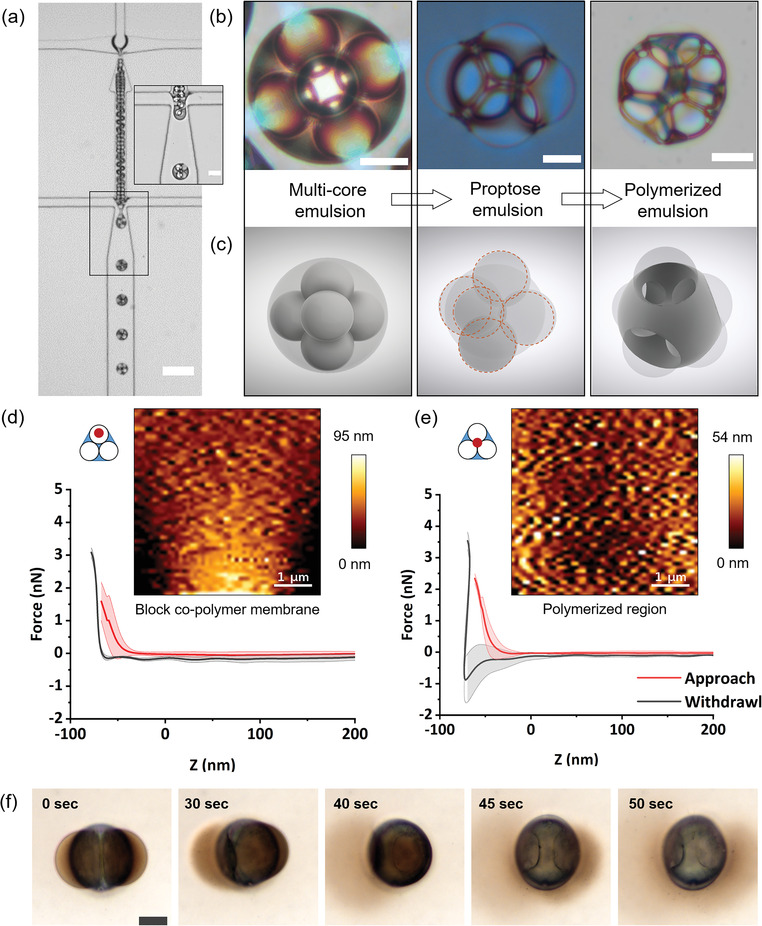
Emulsion‐templating and mechanical characterization. a) High‐speed camera imaging of the 5‐in‐1 multicore emulsion production (scale bar: 200 µm). Inset showing the encasing of the aqueous droplets to form multicore emulsions (scale bar: 20 µm). b) Photo‐polymerization of the 5‐in‐1 multicore emulsion to a polymeric microemulsion with intact membranes (scale bar: 20 µm). c) Computational rendering of the emulsion transition from unpolymerized to polymerized multicore emulsion. d) AFM force‐deformation spectra and the indentation profile of the block copolymer bilayer region of the polymerized emulsion (*n* = 10). e) AFM force‐deformation spectra and the indentation profile of the polystyrene region of the polymerized emulsion (*n* = 10). 3‐in‐1 polymerized emulsion schematic with a red dot represents the area of indentation in the case of the bilayer region and polymerized region. f) Osmotic stress‐induced release of gold nanoparticles from 2‐in‐1 polymerized emulsion.

**Figure 4:**
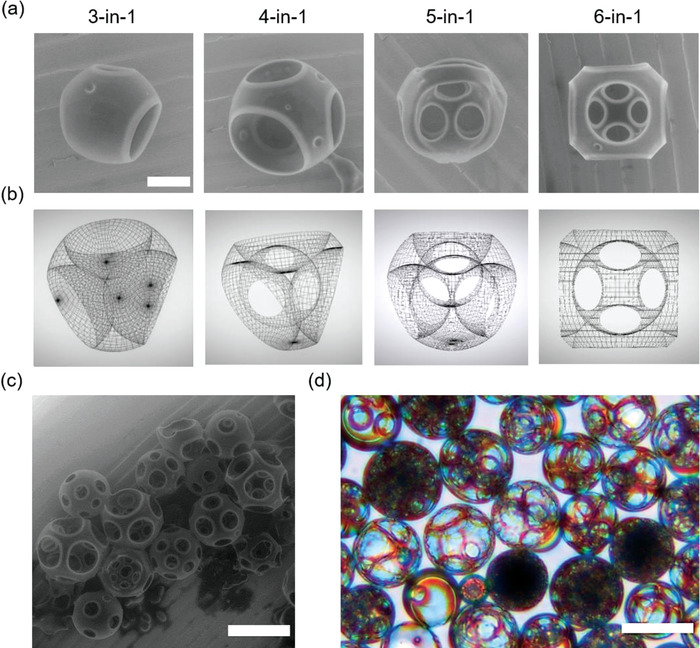
Architectures of skeletal particles with varying pore sizes. a) Scanning electron micrographs of polymerized emulsions to yield corresponding geometries from 3‐in‐1, 4‐in‐1, 5‐in‐1, and 6‐in‐1 multicore emulsions (scale bar: 20 µm). b) Computational renderings of 3‐in‐1, 4‐in‐1, 5‐in‐1, and 6‐in‐1 multicore emulsions show compartments and pore structures, otherwise hidden. c,d) SEM and color images of skeletal particles extracted from various multicore emulsions (scale bar: 100 µm).

Effective stabilization of the produced emulsions is achieved through the inclusion of 0.5 wt% F108 surfactants in both inner and outer aqueous phases during their production. Previous research suggests that specific concentrations of block copolymer surfactants drive the flow‐out of thinner films and even surfactant black films.^[^ ^13^, ^21^ ^]^ At 0.5 wt%, the concentration of the F108 surfactant is sufficient to cover the entire surface of the emulsion, thus allowing for stable emulsion production as well as assisting in dewetting by forming stable membranes and preventing any coalescence.^[^ ^22^ ^]^ For this to occur, the produced emulsions should remain stable and retain their inner cores during the transition period. The block copolymer surfactant used in this study indeed retained the emulsion integrity with >95% are stable (with intact inner cores, retaining their size as well as number of inner cores). This bolsters the idea of using F108 surfactant for not only stabilizing the oil–water interface but also the water–oil interface, which could potentially form membranes. Figure 2b depicts the gradual transformation of multicore emulsions to protruded‐like structures with intact block copolymer membranes—from now “proptose emulsions.” The transition of multicore emulsions to proptose emulsions can be explained via capillary force‐driven film flow and the coupled formation of ultra‐thin block copolymer membranes.^[^ ^13^ ^]^ Complementing previous understanding, density plays an important role in accelerating the overall maturation process, i.e., from spherical multicore emulsion to structured proptose emulsion. The density of the inner aqueous droplets is higher than that of the styrene phase (*ρ*: 909 kg m^−3^). At equilibrium, this allows for spontaneous descent of the inner aqueous droplets within the styrene phase to the bottom of the emulsion (Figure 2b‐1–3), increasing the film pressure and amplifying initial flow. At lower concentrations of surfactant (0.1 wt%), this gravity‐driven pressure on the styrene phase resulted in the coalescence of the inner cores and an eventual collapse of the entire emulsion. This undesired case is mitigated by the applied block copolymer at optimal concentration of 0.5 wt%. However, a constant gravity‐driven compression force of inner aqueous droplet interfaced with block copolymer monolayer results in thinning of the styrene phase present between two aqueous layers (between two inner aqueous cores and also between the inner aqueous core and the outer aqueous solution) (Figure 2b‐3). This first leads to layer thinning, then nucleates the formation and propagation of the block copolymer membranes.^[^ ^23^, ^24^ ^]^ Figure S2a in the Supporting Information depicts a series of maturation snapshots observed in 3‐in‐1 emulsion types. As opposed to an acorn‐like dewetting, we detect a nucleated film formation. This suggests that the maturation, after the gravity‐driven nucleation, is a combination of dewetting and solvent evaporation mechanisms, but of no specific order (Figure 2b‐4–6 and Video S2, Supporting Information). The successful transformation of multicore emulsions to proptose emulsions is marked and stabilized by intact block copolymer membranes (inset Figure 2d and Video S3, Supporting Information). The exceptional stability of the block copolymer membranes can be explained by tight packing and the proven ability of the block copolymer surfactant system to withstand high pressures.^[^ ^21^ ^]^ Such stable packing allows for intact inner aqueous cores with a mere skeletal phase of styrene interfacing their junctions as shown in Figure 2c, 2‐in‐1 proptose emulsions and 3‐in‐1, 4‐in‐1, 5‐in‐1 and 6‐in‐1 in Figure 2d inset (the brilliant colors of the different proptose emulsions are presented in Video S3, Supporting Information). These dewetted emulsions remained stable and had higher density compared to their parent emulsions, due to the loss of excess styrene phase (Figure S2b, Supporting Information).

### 2.2 Interface Polymerization and Mechanical Properties of Proptose Emulsions

**Figure** **3**a emphasizes the reliability and precision capabilities of the microfluidic design used here to produce emulsions with uniform diameter, even with multiple inner aqueous cores, in this case with five inner cores (Video S4, Supporting Information). The diameters of both W/O (26.4 ± 1.8 µm, RSD: 4.07) and 5‐in‐1 W/O/W (63.3 ± 1.7 µm, RSD: 2.75) emulsions have a very narrow size distribution with a low coefficient of variance (Figure S3, Supporting Information). Figure 3b,c shows a complementary view of both experimental as well as rendered images of 5‐in‐1 multicore emulsion transformations. In Figure 3b, the left panel depicts a color image of a 5‐in‐1 multicore emulsion, the brilliant colors of the emulsions are due to the refraction of light as it passes through fluids of different refractive indices. The same is true with proptose emulsions as seen in Figure 2c,d inset as well as in Figure 3b. More interestingly, the styrene present in these emulsions can be photo‐polymerized. Using an UV‐based photoinitiator, 5‐in‐1 emulsions are polymerized after their transformation to proptose emulsions. In the third panel of Figure 3b, an example of the polymerized proptose emulsion is presented. For better understanding to the reader, computational renderings of the emulsions shown in Figure 3b are presented in Figure 3c. Surprisingly, the block copolymer bilayer remained stable even after polymerization. This observation has implications in the field of polymersomes where membrane intactness and stability are of utmost importance for various applications.^[^ ^25^ ^]^ We systematically quantified the mechanical stability of these membranes using AFM‐based nanoindentation studies. This technique is frequently employed in studying the mechanical properties of various thin films.^[^ ^26^, ^27^ ^]^ We performed the force‐deformation experiments, Figure 3d,e shows the average curve obtained for the block copolymer membrane region and polymerized region of the polymerized proptose emulsions (*n* = 10), respectively. While the approach curve is gradual with no jump interactions (attraction force between the cantilever and the indenting surface) for both the regions, the withdrawal curve shows adhesion (max. value: 1.82 ± 0.3 nN) for the polymerized region toward the cantilever. A graph plotting the adhesion values observed for the polymerized region at different force interactions can be seen in Figure S4 in the Supporting Information. The minimal adhesion of the surfactant bilayer region compared to the polymerized region can be explained through the presence of polyethylene oxide (PEO) grafts in the block copolymer molecules facing toward the cantilever. The tight packing of the surfactants at the interface allows for a flexible film with strong steric repulsions between the head groups (PEO chains) and lower adhesion toward the cantilever.^[^ ^28^ ^]^ Any perceived interactions are repelled and thus contribute to a minimal adhesion. However, the same cannot be said for the polymerized region, lack of surfactant and a stiffer polystyrene surface account for indentation and thus adhesion. The block copolymer membrane withstood indentation force of at least 15 nN before rupturing at 20 nN (Video S5, Supporting Information) with an averaged elastic modulus of 6 MPa compared to the polymerized region, 1.2 GPa observed here and elsewhere.^[^ ^29^, ^30^, ^31^ ^]^ The average elastic modulus values of the block copolymer membrane is close to the lipid bilayer region and far from the values obtained for polystyrene thin films, suggesting the absence of polystyrene within these membranes.^[^ ^31^, ^32^ ^]^ Further confirmation is attained via confocal microscopic imaging of the polymerized emulsion. Under UV excitation, polystyrene signal emission is not observed along the intact block copolymer membrane of the polymerized emulsion (Figure S5, Supporting Information).

In addition to force indentation, we have performed osmotic stress‐induced rupturing of the block copolymer membranes (in the presence of hypotonic solution). Similar to lipid vesicles, polymersomes produced from block copolymer self‐assembly can undergo osmotic inflation or deflation.^[^ ^33^, ^34^ ^]^ In Figure S6 in the Supporting Information, we show that proptose emulsions produced from 2‐in‐1 multicore emulsions can undergo deflation (in the presence of hypertonic solution) similar to polymersomes while still retaining their shape, thanks to dynamic dewetting. Similarly, these proptose emulsions can also undergo inflation (in the presence of hypotonic solution) resulting in the collapse of the inner cores. These results suggest that the membranes are permeable to water and are affected by osmotic pressure similar to lipid bilayers. One of the potential applications that can be achieved using osmotic pressure imbalance is cargo storage and release. We demonstrated this via osmotic shock‐induced release of encapsulated gold nanoparticles. 2 wt% water‐dispersible gold nanoparticles solution is used as IA to produce 2‐in‐1 emulsions similar to the procedure described above. Thus, produced emulsions are UV polymerized to perform inflation experiments. In Figure 3f, snapshots of the membrane rupture and release of encapsulated gold nanoparticles from the two inner cores of a 2‐in‐1 polymerized emulsion can be seen (Video S6, Supporting Information). Within a few seconds, the first compartment is ruptured, followed by the second resulting in the complete release of the gold nanoparticles. This experiment not only provides basis for understanding the mechanics of the F108 block copolymer membrane but also sheds light on potential applications such as cargo delivery. After the rupture and release process, the structured nature of the particles (two distinct cavities) can be seen. This suggests that the polymerized proptose emulsions can be further transformed into mere skeletal structures that could result in sophisticated material geometries.

### 2.3 Multicavity Microparticle Geometries with Structural Coloring

**Figure** **4** depicts the scanning electron microscope (SEM) micrographs and their corresponding computational renders for bilayer extracted polymerized proptose emulsion types 3‐in‐1, 4‐in‐1, 5‐in‐1, and 6‐in‐1. A complete translation of 2‐in‐1 multicore emulsions to polymerized alternatives with an intermediate proptose emulsion stage with a conversion efficiency of ≈74.5% is shown in Figure S7 in the Supporting Information. Unlike the SEM micrographs, the wireframe renderings allow for in‐depth information on the precise position and interfaces between the, now nonpresent, inner aqueous cores. The most interesting observation is that the adjacent inner aqueous cores are more compressed and deformed, squeezing styrene in‐between them while forming block copolymer membranes that can be extracted after polymerization (Figure 2b‐3). Indeed, after bilayer extraction, this phenomenon has translated into pores between the aqueous inner core compartments (Figure 4a). The pores that are otherwise hidden in the SEM micrographs are observable in Figure 4a for 5‐in‐1 and 6‐in‐1 and all of the emulsion types in the computational renders (Figure 4b and Video S7, Supporting Information). The SA:V graph is plotted to specify the increase in surface area of these structured microparticles for a potential to be altered into functional surfaces for various applications (Figure S8, Supporting Information). The plot suggests that the increase in the SA:V ratio is directly proportional to the number of pores formed after the carving. For better understanding, an example of computationally rendered 5‐in‐1 multicore emulsion before and after the carving can be seen in Figure S9 in the Supporting Information. Furthermore, we found that these emulsions are optically active, thanks to the higher refractive index of the polystyrene. While multicore emulsions pose a challenge to stability under strain, their alternatives—proptose emulsions and their polymerized counterparts, are more stable and can be exploited for structural coloring as well as other photonic applications. An example of the light‐scattering properties of the polymerized emulsions under different illumination conditions can be seen in Figure S10 and Videos S8 and S9 in the Supporting Information. While the self‐assemble layers are hard to visualize under bright‐field conditions (Figure S10, left panel, Supporting Information), polarized light reveals the highly ordered nature of the self‐assembled polymer surfactant chains (Figure S10, middle panel, Supporting Information). In addition to this, the color image (Figure S10, right panel, Supporting Information) reveals the spectacular light dispersion properties within the skeletal structures (Figure 4d and Figure S10, Supporting Information). This is possible because of the changing refractive index resulting in refraction and optical interference at the surface as predicted in earlier works.^[^ ^35^, ^36^ ^]^

## 3 Conclusion

Defined processes and confined spaces in microfluidics can result in the generation of precise geometries.^[^ ^37^ ^]^ In this work, we exploited the swelling behavior of PDMS‐based microfluidic channels to architect tunable (size and number of cores) multicore emulsions, all without the need for glass microfluidics. Our results suggested that styrene‐based PDMS swelling is reversible and the microfluidic chip can be reused. This methodology allowed to generate a diverse set of multicore emulsion production with ease. Thus, produced emulsions are not only uniform in size and contents (inner core count) but also remained stable without coalescence (≈95%). The usage of a block copolymer surfactant during the generation of these emulsions allowed for the stable conversion of multicore emulsions to proptose emulsions (≈83%) via gravity‐driven dewetting mechanism. The formation of block copolymer membranes ripped the structures where liquid oil skeletons are pulled into shape by membrane elasticity. Further, polymerization of the oil phase was possible while preserving the shape of the skeletal oil phase and creating the most unconventional polymer emulsion particles with a highly organized internal pore structure (≈74.5%). The elastic modulus measurements and confocal imaging proved the absence of polystyrene within the membranes. The presence of stable block copolymer membranes can provide opportunities for applications in the form of multistep catalysis and molecular transport across different compartments of the polymerized emulsions.^[^ ^6^ ^]^ We have shown cargo release possibilities of these polymerized emulsions that can be exploited for cargo delivery purposes in conjunction with biodegradable polymers instead of polystyrene for wider applicability. With the introduction of an inverted emulsion‐based methodology for complex emulsion generation, dewetting‐assisted interface templating production is made more accessible for widespread adoption.^[^ ^38^ ^]^ Thus, generated particles can be model systems for photonic effects, but also the analysis of the physical chemistry of porous materials. The possibility to polymerize such structures and functionalize them with different chemistries opens avenues in creating materials not only with unique architectures but also functionalities.

## 4 Experimental Section

### Microfabrication

Preheated (30 min at 200 °C) 4 in. silicon wafer was spin‐coated (model no. WS‐650MZ‐23NPPB, Laurell Tech. Corp) as per the specifications provided by the manufacturer at 23 °C. Pre‐ and post‐baking steps are performed at 65 °C for 3 min and 95 °C for 9 min, respectively. After 8 s of UV exposure (using Kloe photolithographic instrument (model no. UV‐KUB 2) through film mask design), the wafer was post‐baked at 65 °C for 2 min and 95 °C for 7 min. Followed by a 2 min washing step with the developer solution and later with isopropanol, the wafer was baked at 200 °C for 2 h before performing overnight surface passivation with 50 µL of 1*H*,1*H*,2*H*,2*H*‐perfluoro‐decyl trichlorosilane in a desiccator. PDMS:curing agent (10:1) mixture was thoroughly mixed and degassed for 30 min at 150 millibars low pressure. The degassed mixture was poured on top of the surface‐passivated wafer and cured at 90 °C for 3 h. Thus, crosslinked PDMS was pealed from the master mold and diced into small pieces under a clean hood. A 1 mm biopsy puncher ((Kai Europe GmbH) was used to create the inlets and outlets. Finally, plasma cleaned (at 600 mbar for 1 min) (Plasma Cleaner PDC‐002‐CE, Harrick Plasma) glass coverslips and diced PDMS chips were bonded to form the microfluidic chip. These chips were further heated at 60 °C for 2 h to complete the bonding process and retention of the hydrophobic surface.

### PDMS Swelling Studies

Styrene‐induced swelling of PDMS was critical for the production of multicore emulsions in this work. To characterize the swelling behavior and the role of channel constriction in reducing the W/O droplet size, a series of experiments were performed. Initially, the reversible nature of the PDMS swelling was studied by flooding the 1st cross‐junction (W/O droplet forming junction) with water through the IA inlet. This was followed by completely replacing the water with styrene and later with water again. This process was repeated at least five times from which the change in thickness was measured (*d*) to calculate the degree of swelling (SD)

(1) $$\mathrm{Swelling}\;\mathrm{degree}\left(\%\right)=\left(d_{\mathrm{swollen}}/d_{\mathrm{dry}}-1\right)\times 100$$

### Surface Passivation

Surface treatment of the double emulsion microfluidic design was performed to produce stable double emulsions (Figure S11, Supporting Information). Layer‐by‐layer deposition of charged polymeric solutions was pumped in the direction of the outlet to the outer aqueous solution—to render the hydrophobic PDMS surface hydrophilic. Initially, a 2:1 mixture of H_2_O_2_‐HCl solution was pumped for 30 s, followed by 10 wt% of Polydiallyldimethylammonium chloride (PDADMAC) solution for 2 min and later by 5 wt% of polystyrene sulfonate (PSS) solution for another 2 min. At the end of each step, MilliQ water was pumped for 30 s to remove excess material. Thus, coated chip was ready to be used after 30 min.

### Multicore Emulsion Production

Surface‐treated microfluidic chip was used to produce these emulsions. To form multicore emulsions, the swelling property of the PDMS in the presence of styrene was exploited to narrow the channels at the first junction (at its lowest dimension) where water‐in‐oil (W/O) droplets were created. To produce W/O droplets, an inner aqueous solution (IA) containing 0.5 wt% of F108 surfactant in MilliQ water or gold nanoparticles in MilliQ water was pumped through the inlet. Similarly, styrene containing 5% 1‐octanol was shown to dewet spontaneously in the presence of lipids/surfactants, thus could assist in the dewetting process.^[^ ^5^ ^]^ Furthermore, 1‐octanol allowed for good dispersion of molecules with polar moieties that were otherwise nondispersible in styrene (e.g., lipids and functionalized catalysts for future applications) was pumped towards the 1st cross‐junction to produce W/O droplets.^[^ ^4^ ^]^ Because styrene induced PDMS swelling, the cross‐junction was left with a tiny opening toward the 2nd cross‐junction. A careful alteration of the flow pressures resulted in controlling the number of aqueous droplets that get encased to produce multicore emulsions at the 2nd junction where the outer aqueous solution containing 0.5 wt% F108 surfactants was pumped. Since styrene was used as the oil phase, thus produced droplets could be polymerized to yield polymeric styrene microspheres.

### UV‐Emulsion Polymerization

Multicore emulsions (prepared with styrene:octanol (95:5%) oil phase dissolved with 0.5 wt% BAPO) transitioned to proptose emulsions were placed under UV illumination (395–400 nm, custom‐made device as described in Materials section) for 4 h for complete polymerization of styrene. These polymerized emulsions were imaged or washed with methanol via centrifugation before imaging using SEM (JSM‐7500F, JEOL).

### Osmolarity Studies

Proptose emulsions were subjected to either hypertonic or hypotonic solutions to understand the role of osmotic pressure on the structural integrity of the emulsions. For this, proptose emulsions were transferred to a chamber made of microscopic glass slides fitted with imaging spacers and covered with a glass coverslip leaving an opening for solution injection. NaCl solutions were injected to achieve +100 mOsm and +200 mOsm hypertonic conditions within the chamber, resulting in the deflation of the proptose emulsions. In the case of inflation, proptose emulsions or polymerized 2‐in‐1 emulsions encapsulated with gold nanoparticles were subjected to hypotonic solution.

### Nanoindentation Experiments

Mechanical properties of the polymerized proptose emulsions were performed in aqueous suspension using an AFM (JPK NanoWizard 3 from JPK, Berlin, Germany) equipped with qp‐BioAC CB1 cantilever (uniqprobe, NanoWorld AG) with a spring constant of 0.2 N m^−1^, 7 µm tip height, and 8 nm tip radius. Sample preparation included a coating of the glass coverslip with a positively charged poly‐l‐ornithine solution for 20 min, followed by washing with Milli‐Q water. Particle suspension was allowed to settle down for 15 min before scanning. 3D‐scanner with 15 µm piezo‐driven stroke in the *z*‐direction and 100 µm in the *x*‐ and *y*‐direction was used. 5 × 5 µm size was scanned with different set points at 1, 5, 10, 15, and 20 nN (for the block copolymer membranes) and 1, 2, 3, and 4 nN (for the polymerized zone). Data processing was performed using JPK Data processing software.

### Microscopy

Microfluidic production of multicore emulsions was recorded with a MicroLab 310 camera at full‐frame and ≈3000 fps (Vision Research Inc.) that was connected to a wide‐field Olympus IX73 microscope using an x5 objective in bright‐field transmission mode. Confocal images were acquired with Leica TCS SP8 (Leica Microsystems Inc.) confocal microscope fitted with x20 objective with DAPI excitation and emission collected between 400 and 500 nm. Color images were obtained with Nikon DS‐Fi3 high definition camera fitted to a wide‐field Olympus IX73 microscope using either x5 or x10 or x40 objective in bright‐field transmission mode. In all the cases, the 50 µL of emulsion suspensions were pipetted onto a 0.17 mm glass coverslip fitted with imaging spacers (SecureSeal) for imaging. General image processing was performed using ImageJ/Fiji. Computational rendered emulsions were generated using Vectary and Blender.

### Statistical Analysis

Data presentation and the sample size were included either in the figure captions or the main text. Data were processed using OriginLab Origin 2021b software.

## Conflict of Interest

The authors declare no conflict of interest.

## Supporting information

Supporting Information

Supplemental Video 1

Supplemental Video 2

Supplemental Video 3

Supplemental Video 4

Supplemental Video 5

Supplemental Video 6

Supplemental Video 7

Supplemental Video 8

Supplemental Video 9

## Data Availability

The data that support the findings of this study are available in the Supporting Information of this article.
