# Supplementary material for: Dewetting‐Assisted Interface Templating: Complex Emulsions to Multicavity Particles
Source: Adv Sci (Weinh). 2022 Aug 12;9(29):2203265. doi: 10.1002/advs.202203265 (PMC9561762; doi:10.1002/advs.202203265)
Supplement: Supplementary file 1 — Supporting Information [file ADVS-9-2203265-s008.pdf]

Supporting Information

Dewetting-assisted interface templating: complex emulsions to multicavity particles

Naresh Yandrapalli, Markus Antonietti*

Max Planck Institute of Colloids and Interfaces, Department of Colloid Chemistry, Am Mühlenberg 1, 14424 Potsdam, Germany

Materials

All materials were used as purchased unless noted otherwise. 1-octanol (99 %, Sigma Aldrich), phenylbis(2,4,6-trimethylbenzoyl)phosphine oxide (BAPO initiator, 97%, Sigma Aldrich), Synperonic® F 108 surfactant (Sigma Aldrich). Polydimethylsiloxane (PDMS) and curing agent were obtained as SYLGARD® 184 silicone elastomer kit from Dow Corning. 1H,1H,2H,2H-Perfluorodecyltrichlorosilane was purchased from abcr GmbH. 30 nm Gold nanoparticles, Poly(diallyldimethylammonium chloride (PDADMAC) and poly(sodium 4-styrenesulfonate (PSS) were obtained from Sigma Aldrich. SU8 2050 (Microchem Inc.), Silicon wafer (Siegert Wafers), SU8 developer solution (Microchem Inc.) Styrene (99 %, Sigma Aldrich) was passed through alumina column to remove the inhibitor before use. MilliporeSigma™ Poly-L-Ornithine solution (0.01%) was purchased from Fisher scientific. 50W LED chips (Foxpic High Power 50 W LED Chip Bulb Light DIY White 3800LM 6500 K) and 30 W UV chip (Fdit, 395-400 nm UV LED chip) were connected to a self-made circuit and cooling system. For fluid flow control, four-channel pressure devices are used (MFCS-EZ, Fluigent Inc.). Isotonic conditions are maintained unless otherwise mentioned.

Supplementary Figures

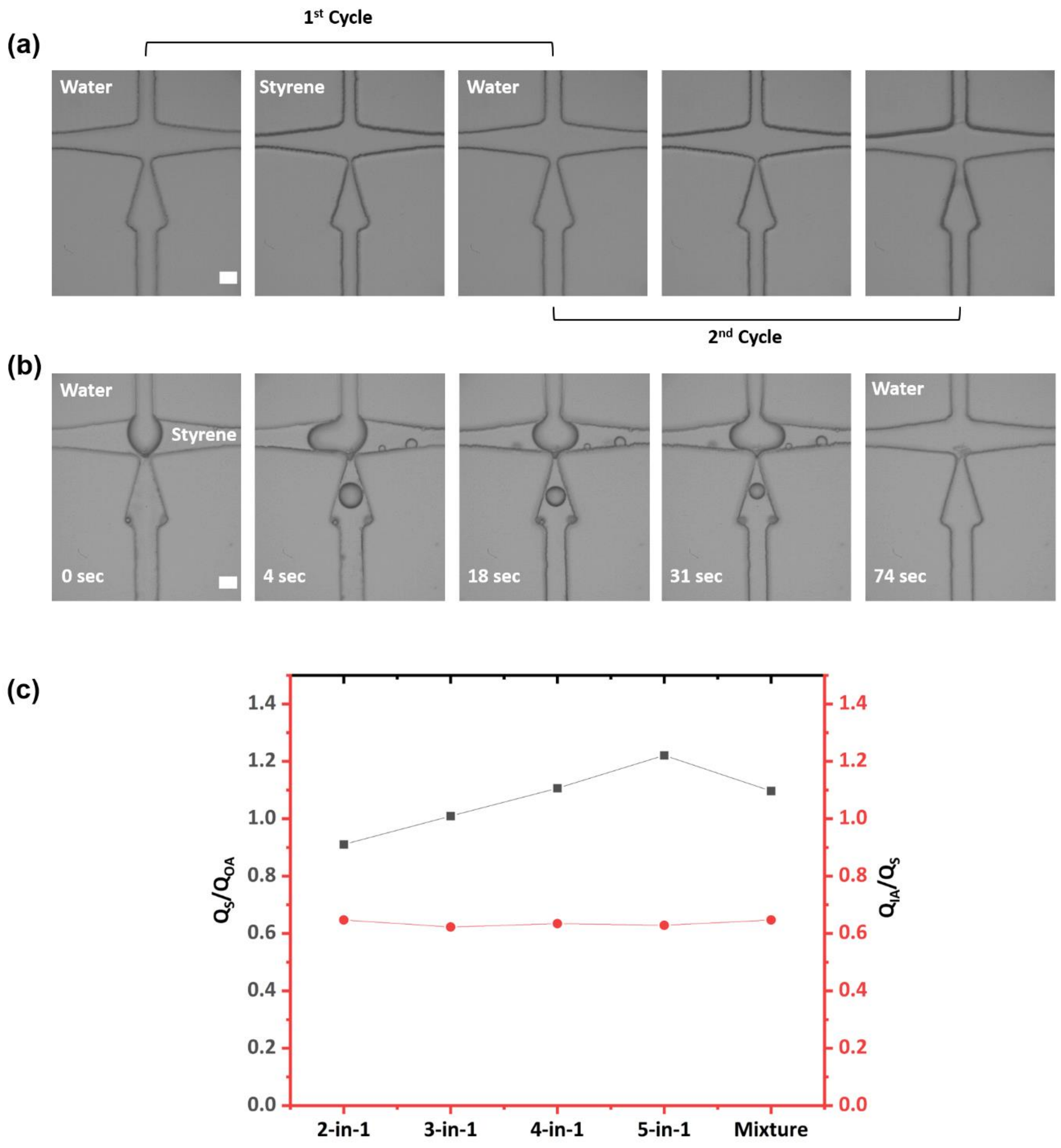

Figure S1. PDMS swelling-based multi-core emulsion production. (a) Reversible PDMS swelling cycles are shown from left to right, in the presence of water (de-swelling) or styrene (swelling) resulting in wider or narrow microfluidic channels, respectively (Scale bar – 50 µm). (b) Snapshots of PDMS swelling as well as a gradual decrease in W/O droplet size at constant fluid flow rates (IA - 40 and S – 50 mbar) (Scale bar – 50 µm). (c) The fluid flow of the outer

aqueous solution was gradually tuned to produce multi-core emulsions of different types at constant inner aqueous flow to the styrene phase ratio.

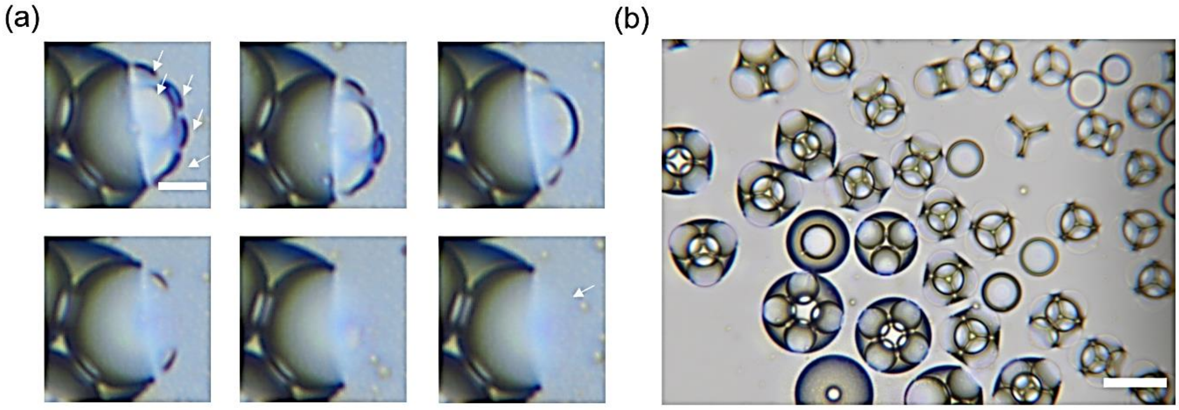

Figure S2. Multi-core emulsions to Proptose emulsions. (a) Snapshots of time-lapse (left to right) showing the dewetting of styrene phase in the form of multiple droplets (white coloured arrows), leaving a surfactant bilayer at the end (Scale bar – 10 µm). (b) The gradual shift in plane of focus of the emulsions from left to right emphasizes the change in their density, from lower (more styrene phase in multi-core emulsions) to higher (low styrene phase in proptose emulsions).

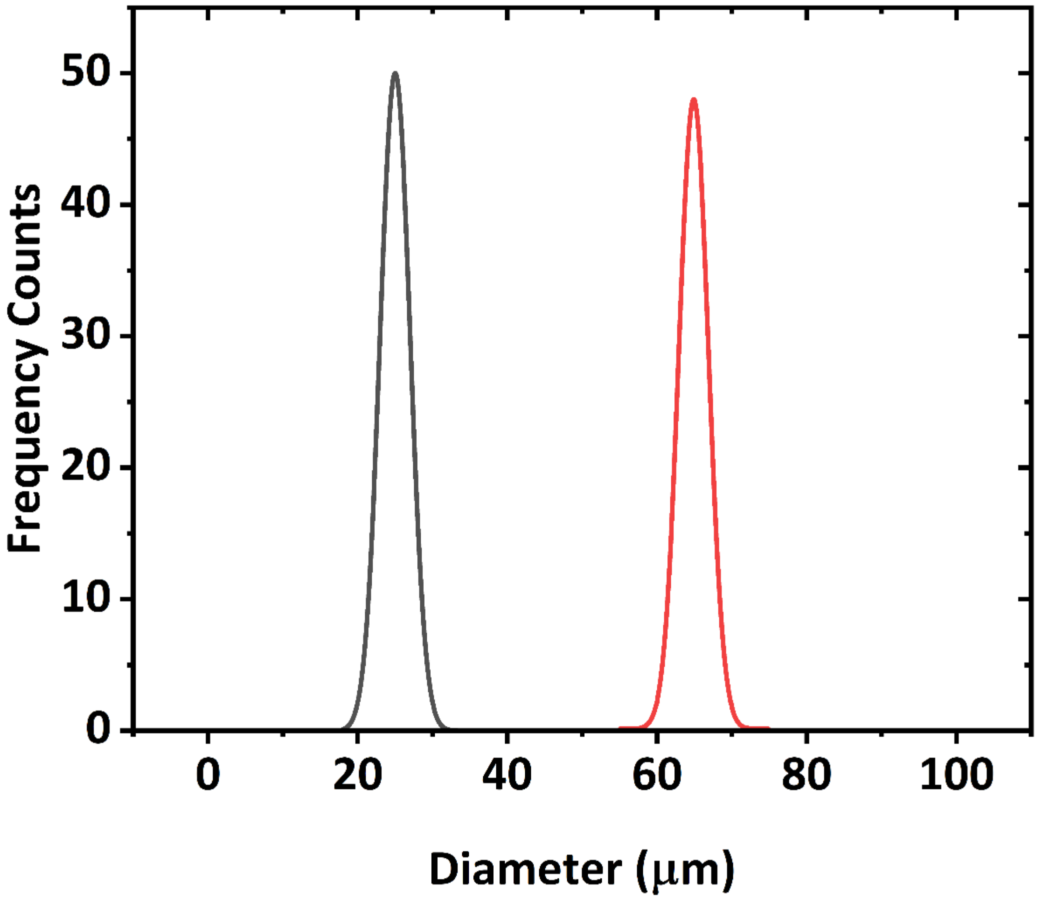

Figure S3. Plot showing the narrow size distribution of the inner aqueous droplets (black peak) (Mean diameter is 26.4±1.8 µm, RSD – 4.07) encased to form 5-in-1 multi-core emulsion (red peak) (Mean diameter is 63.3±1.7 µm, RSD – 2.75) (n = 50).

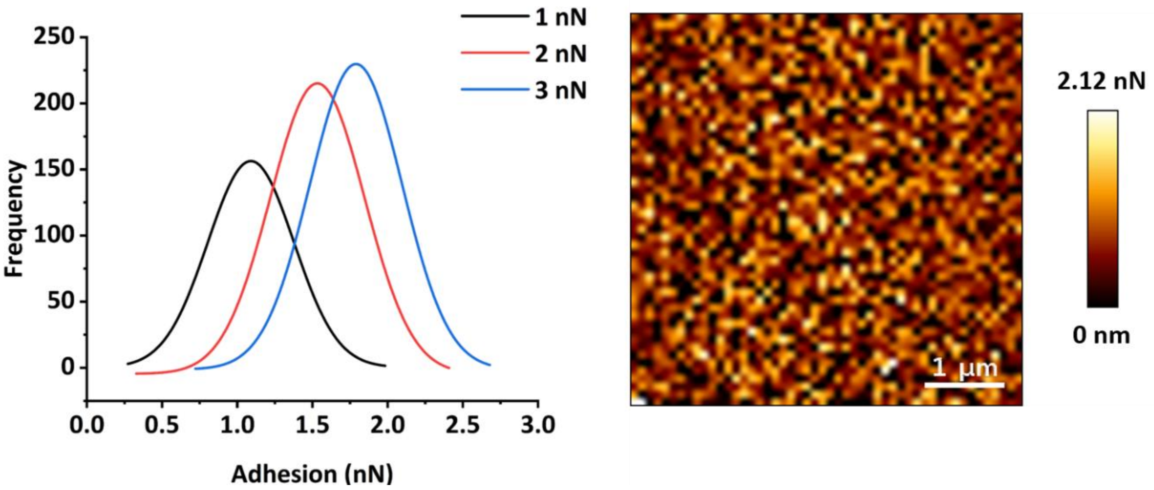

Figure S4. Histograms of adhesion values plotted from different force-deformations performed on the polymerized region of the emulsion (inset showing the adhesion image at 3 nN).

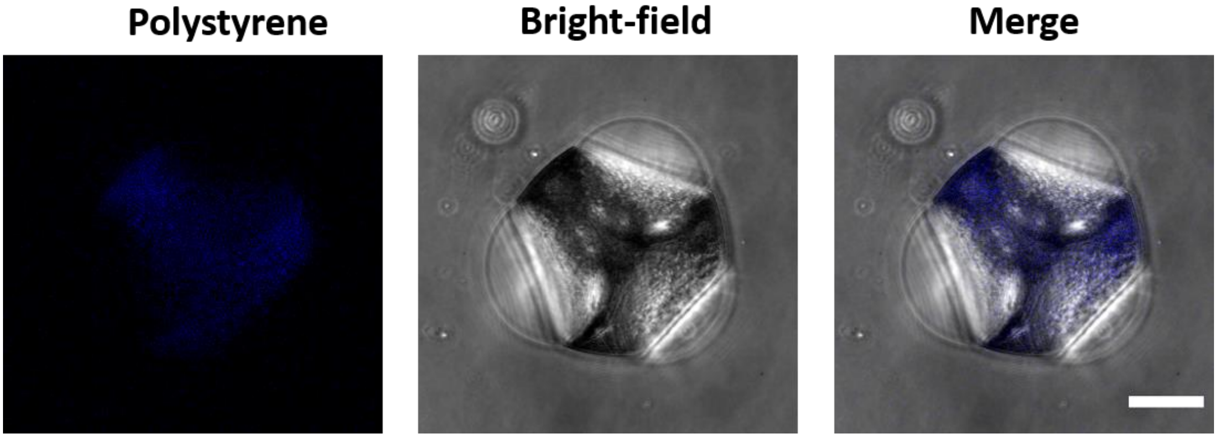

Figure S5. Confocal microscopic images of polymerized 3-in-1 emulsion with intact block copolymer membranes show no emission under UV excitation that is observed typically for polystyrene. (Scale bar – 25 µm).

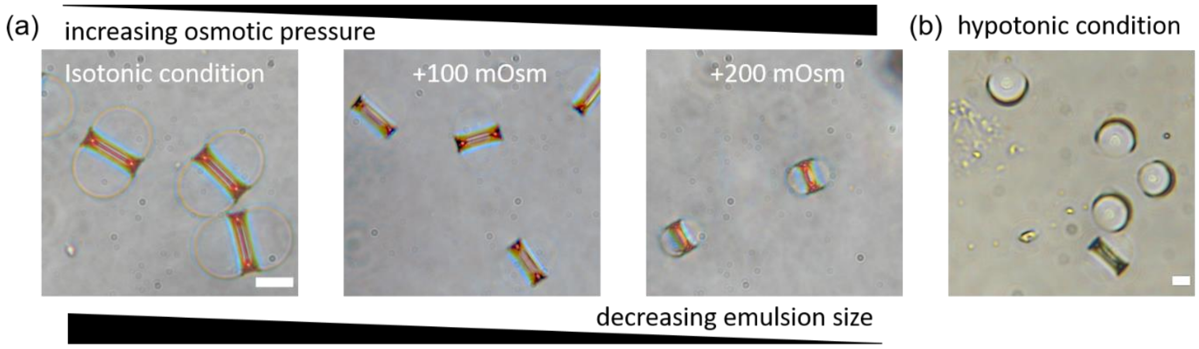

Figure S6. **Effect of osmolarity change in the particle geometries.** (a) In the presence of hypertonic solution, proptose emulsions gradually reduced their size from 63±2 µm (isotonic condition) to 41±1 µm (+100 mOsm) to 26±1 µm (200 mOsm). (b) Under hypotonic condition, the inner cores of the emulsion collapse to give one single-core double emulsion. Scale bar – 25 µm

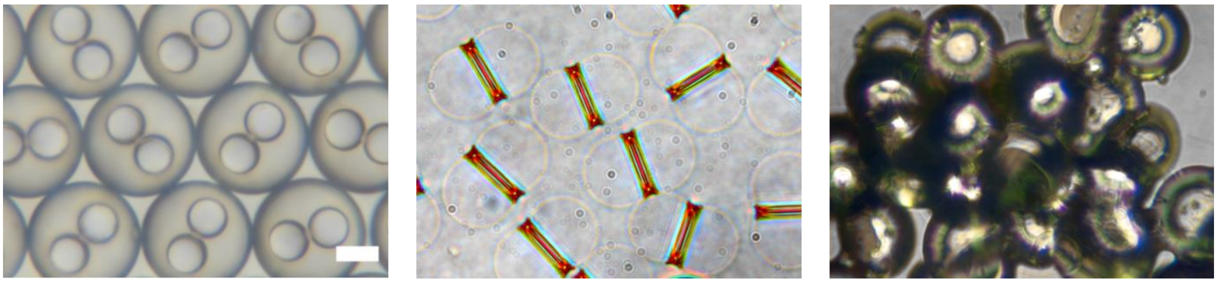

Figure S7. The complete transition of 2-in-1 multi-core emulsions to proptose emulsions and finally into their polymerized multicavity particles is presented from left to right. Scale bar – 25 µm.

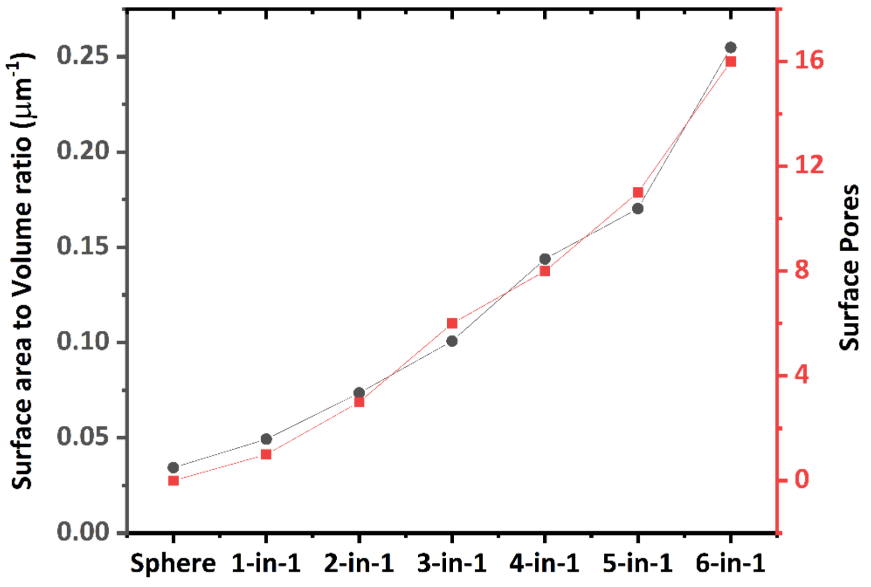

Figure S8. Plot showing the increase in surface area to volume ratio (SA:V) (grey) as well as the surface pores (red) of polymerized emulsions derived from an increasing number of inner aqueous cores.

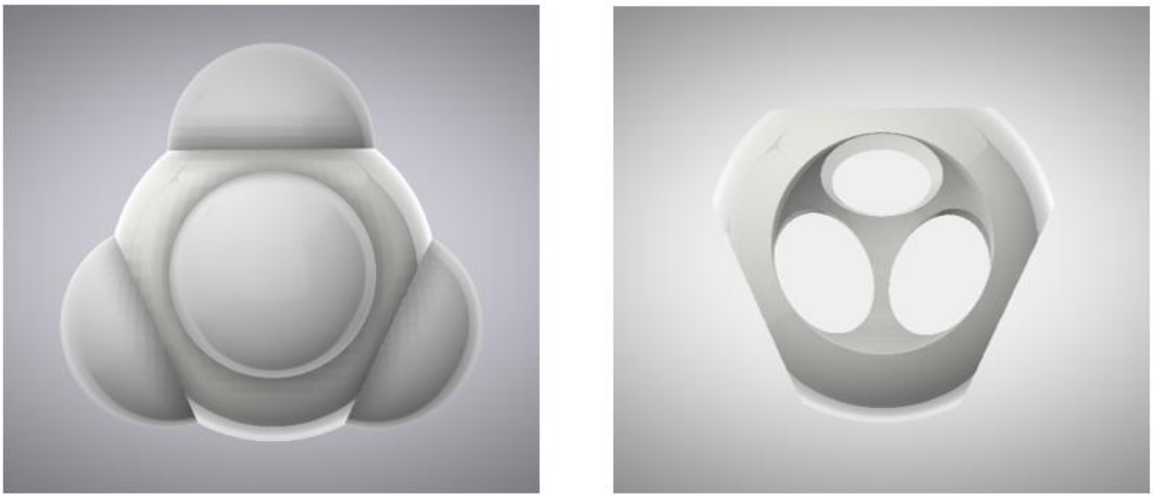

Figure S9. Computational rendering of 5-ni-1 multicore emulsion and its corresponding multicavity particle.

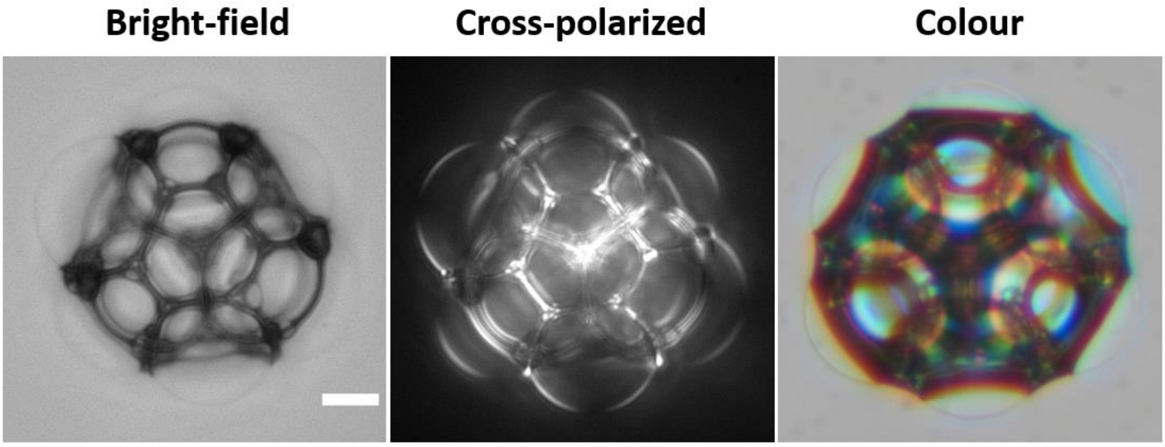

Figure S10. Multi-core emulsion under different lighting conditions. Left panel showing the bright field image, middle panel showing the same emulsion under cross polarization illumination – represents the highly structured nature of the block copolymer membranes and the polymerized styrene. The right panel presents the tru to colour images suggesting the light scattering-induced structural colouration. Scale bar corresponds to 20 µm.

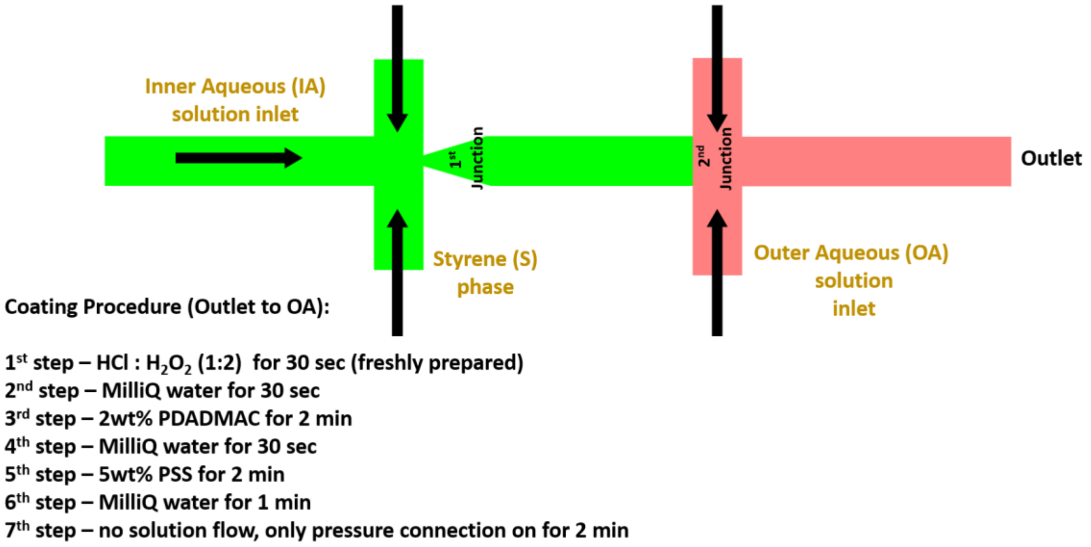

Figure S11. Surface passivation. Schematic showing the respective inlets for the solutions pumped into the microfluidic chip. The bright green region of the chip represents the hydrophobic region of the chip where water-in-oil droplets are formed at the 1st junction and the red region presents the hydrophilic region (after surface passivation). Where multi-core emulsions are formed. All the steps required to achieve the hydrophilic region is also presented.

Supplementary Video Legends

Video 1

Microfluidic production of mixed type multi-core emulsions (IA - 66 mbar, Styrene – 102 mbar, OA – 93 mbar), containing aqueous inner and outer solution and middle styrene solution. The image sequence was acquired using a high-speed camera at ~3000 fps. Scale bar corresponds to 200 µm.

Video 2

Time-lapse video showing the conversion of 2-in-1 double emulsions to their corresponding proptose emulsions. Scale bar corresponds to 20 µm.

Video 3

True to colour image sequence of free-floating proptose emulsions obtained from different multi-core emulsion types.

Video 4

Microfluidic production of 5-in-1 type multi-core emulsions (IA - 57 mbar, Styrene – 60 mbar, OA – 80 mbar), containing aqueous inner and outer solution and middle styrene solution. The image sequence was acquired using a high-speed camera at ~3000 fps. Scale bar corresponds to 200 µm.

Video 5

Time-lapse video of surfactant bilayer rupture using AFM-based nanoindentation at 20 nN set point.

Video 6

Gold nanoparticle cargo is released from polymerized 2-in-1 emulsions using an osmotic stress-induced rupture of block copolymer membranes. Scale bar – 25 µm.

Video 7

Computational rendering of polymeric skeletal particle derived from 5-in-1 proptose emulsion.

Video 8

Bright field and Polarized illumination of 6-in-1 polymerized emulsion Z-stack. Scale bar - 20 µm.

Video 9

Z-stack image sequence of 6-in-1 polymerized emulsion. Scale bar – 20 µm.
